# Efficacy and safety of mesenchymal stem cell transplantation for progressive multiple sclerosis: a systematic review and meta-analysis of randomized controlled trials with clinical implications for patient stratification and treatment optimization

**DOI:** 10.3389/fimmu.2026.1861624

**Published:** 2026-07-22

**Authors:** Honghao Guo, Liuting Zeng, Lingyun Sun

**Affiliations:** 1 Department of Rheumatology and Immunology, Nanjing Drum Tower Hospital, Clinical College of Nanjing Medical University, Nanjing, Jiangsu, China; 2 Department of Rheumatology and Immunology, Nanjing Drum Tower Hospital, Chinese Academy of Medical Sciences & Peking Union Medical College, Nanjing, China

**Keywords:** mesenchymal stem cells, meta-analysis, progressive multiple sclerosis, randomized controlled trials, safety

## Abstract

**Background:**

Progressive multiple sclerosis (PMS) is a disabling demyelinating disease characterized by irreversible neurodegeneration. Unlike relapsing-remitting MS, for which many disease-modifying therapies exist, treatment options for PMS are extremely limited. Only two agents (ocrelizumab and siponimod) have modest efficacy, and no effective therapies exist for non-active disease. Mesenchymal stem cell (MSC) transplantation has emerged as a promising strategy due to its immunomodulatory and neurotrophic properties. However, clinical trials have yielded inconsistent results, and prior meta-analyses were limited by mixed MS subtypes and non-randomized studies, leaving uncertainty about MSC’s clinical utility in PMS.

**Objective:**

To rigorously evaluate the efficacy and safety of MSC transplantation in PMS patients through a systematic review and meta-analysis of exclusively randomized controlled trials (RCTs), and to provide evidence-based recommendations.

**Methods:**

Two researchers searched PubMed, Embase, Web of Science, Cochrane Library, and Chinese databases from inception to March 2026. RCTs comparing MSC transplantation with placebo in adult PMS patients were included. Primary outcome: change in Expanded Disability Status Scale (EDSS) score. Secondary outcomes: treatment-emergent adverse events (TEAEs) and serious adverse events (SAEs). Meta-analysis used RevMan 5.4 with random/fixed-effects models. Certainty of evidence was assessed by GRADE. Protocol registered on PROSPERO (CRD420261301154).

**Results:**

Three RCTs (143 patients; 75 MSC, 68 control) were included. MSC therapy did not significantly improve EDSS scores (pooled MD = -0.08, 95% CI -0.38 to 0.22, *P* = 0.61; *I²* = 66%). MSC did not increase TEAEs (RR = 1.08, 95% CI 0.86 to 1.35, *P* = 0.52) or SAEs (RR = 0.79, 95% CI 0.26 to 2.36, *P* = 0.67). GRADE: moderate certainty for TEAEs, very low for EDSS change and SAEs.

**Conclusion:**

MSC transplantation appears safe in PMS patients, with no increased adverse event risk. However, current evidence does not support significant benefit on EDSS scores in unselected patients. Given the very low to moderate certainty, further high-quality RCTs are warranted.

This systematic review and meta-analysis was conducted in strict accordance with the Cochrane Handbook for Systematic Reviews of Interventions (version 6.5) and reported following the Preferred Reporting Items for Systematic Reviews and Meta-Analyses (PRISMA) 2020 guidelines[24, 25]. The study protocol was prospectively registered on the International Prospective Register of Systematic Reviews (PROSPERO, registration number: CRD420261301154) prior to literature screening and data extraction.

## 1 Introduction

Multiple sclerosis (MS) is a chronic inflammatory demyelinating disease of the central nervous system, characterized by immune-mediated myelin damage, axonal loss, reactive gliosis, and subsequent progressive neurodegeneration (1, 2). MS is primarily classified into relapsing-remitting MS (RRMS) and progressive MS (PMS) based on the course. PMS is further divided into primary progressive MS (PPMS) and secondary progressive MS (SPMS) (3). Globally, approximately 2.8 million people are living with MS, with 10–15% of patients presenting initially with PPMS, characterized by persistent deterioration of neurological function from disease onset (3–5). Approximately 70% of relapsing-remitting MS (RRMS) patients will eventually transition to the SPMS stage within 10–15 years after disease onset (6). Primary progressive MS (PPMS) is associated with faster disability progression and a reduced life expectancy (approximately 7 years shorter compared to RRMS) (7), imposing a heavy burden on patients, families, and healthcare systems (5). Unlike RRMS, for which more than 20 disease-modifying therapies (DMTs) have been approved and achieved remarkable success in reducing relapse rates and disease activity, treatment options for PMS remain extremely limited (8). Only two agents (ocrelizumab and siponimod) have received regulatory approval for PMS, with modest efficacy restricted to patients with underlying inflammatory activity, and no effective therapies exist for non-active progressive disease (9).

Over the past few decades, disease-modifying therapies (DMTs) for RRMS have achieved remarkable progress, effectively reducing relapse rates and MRI lesion burden by targeting peripheral immune cells (10). These agents can be broadly categorized into three generations: first-generation injectable therapies (interferon-beta, glatiramer acetate) that modulate immune responses; second-generation oral therapies (fingolimod, teriflunomide, dimethyl fumarate) that inhibit lymphocyte trafficking or proliferation; and third-generation high-efficacy therapies (ocrelizumab, natalizumab, alemtuzumab) that selectively deplete pathogenic immune cells or block their entry into the central nervous system (10, 11). However, treatment options for PMS remain limited (12). The pathophysiological mechanisms underlying PMS are more complex, involving focal and diffuse inflammation in the periventricular grey matter, oxidative stress, mitochondrial dysfunction, and iron accumulation. These factors collectively contribute to irreversible axonal injury and accelerated brain volume loss (13). Therefore, conventional immunomodulatory agents often fail to halt the relentless progression of neurological disability in PMS patients, underscoring an urgent need for novel therapeutic strategies that promote neurorepair and regeneration.

The two currently approved therapies for PMS have distinct mechanisms of action and limitations. Ocrelizumab is a humanized anti-CD20 monoclonal antibody that depletes circulating B cells, the primary mediators of humoral immunity. The phase III ORATORIO trial demonstrated that ocrelizumab reduced the risk of 12-week confirmed disability progression by 24% in PPMS patients compared to placebo, but this benefit was only observed in patients with gadolinium-enhancing lesions on baseline MRI, indicating active inflammation. Common adverse events include infusion-related reactions and increased risk of upper respiratory tract infections (14). Siponimod is a selective sphingosine-1-phosphate receptor 1 and 5 modulator that sequesters lymphocytes in lymph nodes, preventing their migration to the central nervous system. The EXPAND trial showed that siponimod reduced the risk of 3-month confirmed disability progression by 21% in SPMS patients, again with greater efficacy in those with active disease. Adverse effects include bradycardia, first-degree atrioventricular block, and liver enzyme elevations (15). In contrast to these single-target anti-inflammatory agents, mesenchymal stem cells (MSCs) exert pleiotropic effects through both immunomodulatory and neurotrophic mechanisms. MSCs can suppress pathogenic T cells, B cells, and pro-inflammatory macrophages, while secreting neurotrophic factors such as brain-derived neurotrophic factor (BDNF) and glial cell line-derived neurotrophic factor (GDNF) that promote oligodendrocyte differentiation, remyelination, and axonal protection (16–18). This dual mechanism of action makes MSCs a theoretically superior candidate for PMS, which is characterized by both persistent inflammation and progressive neurodegeneration. However, the clinical efficacy of MSC transplantation in PMS remains controversial due to inconsistent trial results.

Mesenchymal stem cells (MSCs) possess potent immunomodulatory, anti-inflammatory, and neurotrophic properties, and mesenchymal stem cell transplantation (MSCT) has emerged as a highly promising candidate therapy for various autoimmune diseases (18). Preclinical studies have shown that MSCs can modulate aberrant immune responses by suppressing pathogenic T cells and regulating antigen-presenting cells, and they secrete cytokines such as brain-derived neurotrophic factor (16, 17). They facilitate oligodendrocyte differentiation, promote endogenous remyelination, and provide axonal protection in a paracrine manner (16, 17). In recent years, several trials have preliminarily explored MSCT in MS patients, suggesting potential clinical benefits in delaying disability progression and improving neurological function, with a favorable tolerability profile (19, 20). However, some animal studies have shown inconsistent results, where MSCT fails to show efficacy (21). This heterogeneity may stem from multiple factors, including differences in MSC sources (bone marrow, adipose tissue, or umbilical cord), dosage, administration routes [intrathecal (IT) versus intravenous (IV)], patient selection criteria, and follow-up durations.

Although several meta-analyses on stem cell therapy for MS have been published, they have been limited by the inclusion of mixed MS subtypes, non-randomized study designs, and insufficient exploration of heterogeneity sources, leading to uncertain conclusions specifically for the progressive population. A 2024 meta-analysis by Vaheb et al. (22) pooled 30 studies, including non-randomized trials and mixed MS subtypes, and reported a significant EDSS reduction only with intrathecal MSC administration. A 2026 meta-analysis by Maji et al. (23) included 11 RCTs with mixed stem cell types (hematopoietic and mesenchymal) and mixed MS phenotypes, finding a non-significant pooled EDSS reduction of 0.35 points. None of these prior meta-analyses exclusively focused on PMS patients or performed a comprehensive GRADE assessment of evidence quality. Therefore, an updated and systematic synthesis of the available evidence restricted to RCTs in PMS patients is required to provide more reliable clinical guidance. Therefore, we conduct this systematic review and meta-analysis of randomized controlled trials to rigorously evaluate the efficacy and safety of MSCT for PMS.

## 2 Methods

This systematic review and meta-analysis was conducted in strict accordance with the Cochrane Handbook for Systematic Reviews of Interventions (version 6.5) and reported following the Preferred Reporting Items for Systematic Reviews and Meta-Analyses (PRISMA) 2020 guidelines (24, 25). The study protocol was prospectively registered on the International Prospective Register of Systematic Reviews (PROSPERO, registration number: CRD420261301154) prior to literature screening and data extraction.

A comprehensive, peer-reviewed literature search was independently performed by two trained researchers across 10 databases from their inception to March 3, 2026, including English databases (PubMed, Embase, Medline, Web of Science, Cochrane Library, ClinicalTrials.gov) and Chinese databases (CNKI, Wanfang Data, VIP, SinoMed). No language restrictions were applied. The detailed search strategies for PubMed and Embase are provided in **Supplementary Table 1**. Reference lists of included studies and relevant systematic reviews were manually screened to identify additional eligible records.

### 2.1 Studies were included if they met the following PICOS criteria

1. Population: Adult patients (aged ≥ 18 years) with a confirmed diagnosis of PPMS or SPMS according to the McDonald criteria (any version).
2. Intervention: Mesenchymal stem cell transplantation, regardless of cell source (autologous/allogeneic, bone marrow/adipose/umbilical cord-derived), dosage, administration route, or infusion frequency.
3. Comparator: Placebo, saline sham injection, or standard of care.
4. Outcomes: Reported at least one of the pre-specified efficacy outcomes [change in Expanded Disability Status Scale (EDSS) score from baseline, change in MRI T2 lesion volume/number) or safety outcomes (treatment-emergent adverse events (TEAEs), serious adverse events (SAEs)].
5. Study design: Parallel-group randomized controlled trials (RCTs).

### 2.2 Exclusion criteria were as follows

1. Studies enrolling patients with mixed MS subtypes (RRMS + PMS) without extractable separate data for the PMS subgroup.
2. Non-randomized studies, case reports, case series, reviews, editorials, comments, animal experiments, or *in vitro* studies.
3. Studies with incomplete outcome data, duplicate publications, or secondary analyses of previously included trials.
4. Studies enrolling pediatric patients (< 18 years old), or patients with concurrent autoimmune inflammatory diseases other than MS.
5. Studies employing a crossover design that reported only pooled post-crossover outcome data without extractable parallel-group data from the pre-crossover phase.

### 2.3 Literature screening, data extraction, and risk of bias assessment

Literature screening, data extraction, and risk of bias assessment were performed independently by two researchers, with any discrepancies resolved through consensus discussion with a third senior researcher. For each included study, the following data were extracted: first author, publication year, country, study design, sample size, patient baseline characteristics (age, gender, MS subtype, disease duration, baseline EDSS score, disease activity status), MSC characteristics (cell source, autologous/allogeneic, dosage, administration route, infusion frequency), follow-up duration, outcome data for efficacy and safety endpoints, ethical approval information, and conflict of interest disclosures.

### 2.4 Risk of bias assessment

The risk of bias of each included RCT was assessed using the revised Cochrane Risk of Bias tool for randomized trials (RoB 2), which evaluates bias across five domains: random sequence generation and allocation concealment, deviations from intended interventions, missing outcome data, measurement of the outcome, and selective reporting of results (26). Each domain was rated as low risk of bias, some concerns, or high risk of bias, with an overall risk of bias rating assigned to each study.

### 2.5 Meta-analysis

Meta-analysis was performed using Review Manager (RevMan) 5.4 software (Cochrane Collaboration, Copenhagen, Denmark). For dichotomous outcomes (TEAEs, SAEs), effect sizes were expressed as risk ratios (RRs) with 95% confidence intervals (CIs). For continuous outcomes (change in EDSS score), effect sizes were expressed as mean differences (MDs) with 95% CIs. Between-study heterogeneity was assessed using the Chi-squared test and quantified with the *I²* statistic. An I² value > 50% was considered indicative of substantial heterogeneity, and a random-effects model (DerSimonian-Laird method) was applied; otherwise, a fixed-effects model (Mantel-Haenszel method for dichotomous outcomes, inverse variance method for continuous outcomes) was used. Sensitivity analysis using the leave-one-out method was performed to evaluate the robustness of the pooled effect estimates.

We planned to perform subgroup analyses to explore potential sources of heterogeneity according to MSC source (bone marrow vs. adipose vs. umbilical cord), administration route (intrathecal vs. intravenous vs. combined), dosage (<2×10^6^ cells/kg vs. ≥2×10^6^ cells/kg), MS subtype (PPMS vs. SPMS), disease activity status (active vs. non-active), and follow-up duration (≤6 months vs. >6 months). Meta-regression was planned to assess the association between continuous variables (age, disease duration, baseline EDSS score) and treatment effect size.

The certainty of evidence for each primary and secondary outcome was assessed following the Grading of Recommendations Assessment, Development and Evaluation (GRADE) methodology, which rates evidence quality as high, moderate, low, or very low based on five domains: risk of bias, inconsistency, indirectness, imprecision, and publication bias (27). GRADE assessments were conducted using GRADEpro GDT software (McMaster University, Hamilton, Canada).

### 2.6 Multi-arm trials and publication bias

For multi-arm trials, pairwise comparisons were created by splitting the control group according to the proportional distribution of participants in each active arm to avoid unit-of-analysis errors. Publication bias was not formally assessed due to the small number of included studies (<10), as recommended by the Cochrane Handbook (24).

### 2.7 Data preparation and missing data handling

For continuous outcomes, if only median and interquartile range (IQR) were reported, we converted them to mean and standard deviation (SD) using the method described by Wan et al. (28). If only the range was provided, SD was estimated as range/4 for sample sizes <15 and range/6 for sample sizes ≥15. For dichotomous outcomes, if event rates were not directly reported, we calculated them from the raw numbers of events and total participants.

When summary statistics were missing from the text, we attempted to extract numerical data from published figures using the WebPlotDigitizer software (version 4.6, https://automeris.io/WebPlotDigitizer/). If data could not be extracted or obtained from the authors upon request, the study was excluded from the quantitative meta-analysis for that specific outcome. For multi-arm trials, we followed the Cochrane Handbook guidance to split the control group proportionally to avoid unit-of-analysis errors, as described in section 2.6.

## 3 Results

### 3.1 Search results

**Figure 1:**
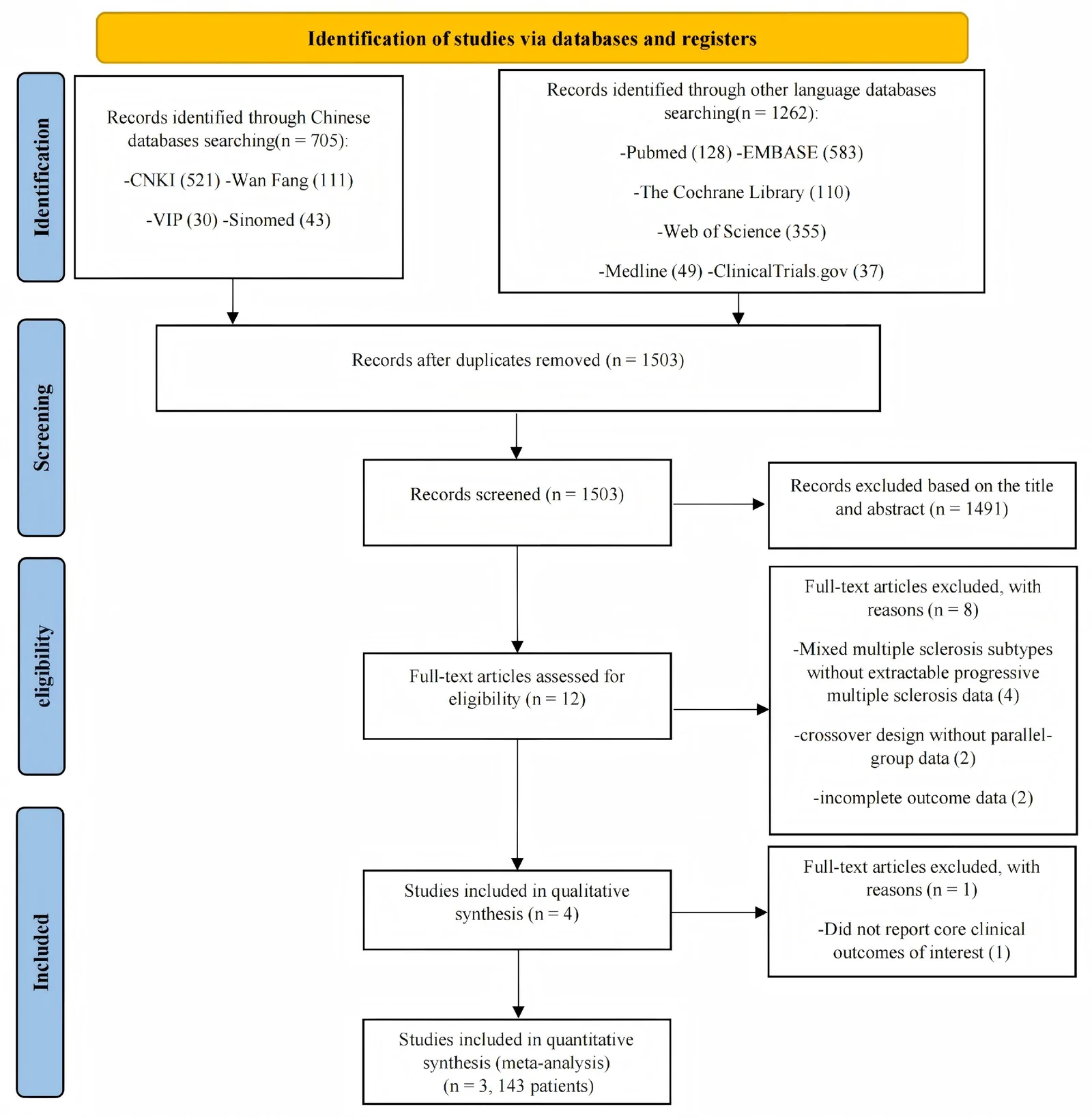
PRISMA 2020 flow diagram of the literature screening process. The final meta-analysis included 3 RCTs, comprising a total of 143 patients with progressive multiple sclerosis (75 in the MSC group, 68 in the control group).

The initial literature search retrieved a total of 1967 records. After removing duplicates, 1503 unique records remained for title and abstract screening, of which 1491 irrelevant records were excluded. The remaining 12 full-text articles were assessed for eligibility against the pre-specified inclusion and exclusion criteria. After full-text review, 8 studies were excluded for reasons including mixed MS subtypes without extractable PMS data, crossover design, and incomplete outcome data (29–36); 1 study was included for qualitative analysis only due to missing quantitative efficacy data (37); and 3 RCTs were finally included in the quantitative meta-analysis (19, 38, 39). The PRISMA flow diagram of the literature screening process is presented in **Figure 1**.

### 3.2 Characteristics of included studies

A total of 3 RCTs enrolling 143 adult patients with PMS (75 in the MSC intervention group, 68 in the control group) were included in this meta-analysis. The key characteristics of the included studies are summarized in **Supplementary Table 2**. All included studies were published between 2018 and 2024, with follow-up durations ranging from 6 months to 12 months. Two studies included both PPMS and SPMS patients (19, 38), while one study exclusively enrolled SPMS patients (39). Two studies used autologous bone marrow-derived MSCs (19, 38), and one study used autologous adipose-derived MSCs (AdMSCs) (39). Administration routes included IT injection, IV infusion, or both, with cell dosages ranging from 1×10^6^ cells/kg to 4×10^6^ cells/kg. All included studies used placebo or sham injection as the comparator, and all were randomized, double-blind, placebo-controlled trials.

### 3.3 Risk of bias assessment

The overall risk of bias assessment results are presented in **Supplementary Figure 1** and S2. Among the 3 included RCTs, 1 study was rated as having an overall low risk of bias, 1 study had some concerns, and 1 study was rated as high risk of bias, mainly due to high risk of selective reporting of results and some concerns regarding missing outcome data. All included studies reported adequate random sequence generation and allocation concealment, and all performed double-blinding of participants, personnel, and outcome assessors. Selective reporting of outcomes was identified as high risk in one study and some concern in another study, while one study had low risk of selective reporting.

### 3.4 Primary outcome: change in EDSS score from baseline

**Figure 2:**
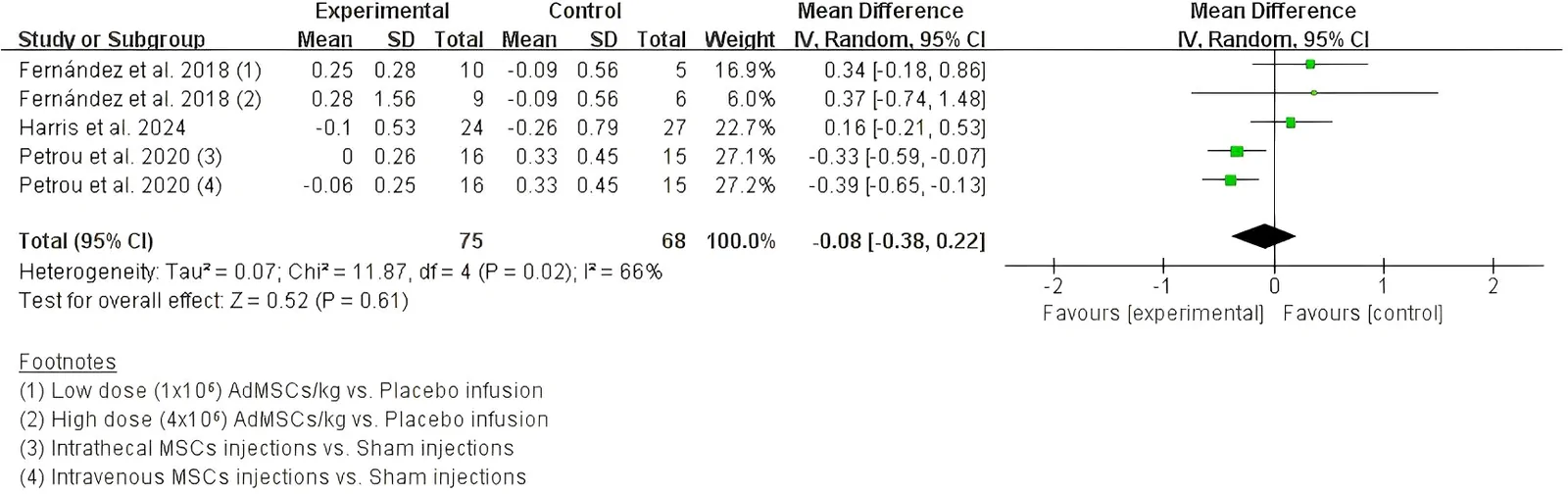
Forest plot of the change in Expanded Disability Status Scale (EDSS) score from baseline after MSC therapy compared with placebo.

All 3 included RCTs (contributing 5 pair-wise comparisons to account for different dosages and administration routes) reported the change in EDSS score from baseline to the end of follow-up. Meta-analysis using a random-effects model revealed that MSC transplantation did not significantly improve EDSS scores in the overall PMS population, with a pooled MD of -0.08 (95% CI -0.38 to 0.22, Z = 0.52, *P* = 0.61). Substantial between-study heterogeneity was observed (*I²* = 66%, *P* = 0.02). Formal subgroup analyses and meta-regression were not performed due to the insufficient number of included studies (n=3), which is below the minimum threshold of 10 studies recommended by the Cochrane Handbook for reliable subgroup analysis (24). The forest plot for the primary outcome is presented in **Figure 2**.

### 3.5 Sensitivity analysis

Sensitivity analysis using the leave-one-out method was performed to assess the robustness of the pooled effect estimate. After sequentially excluding each individual study, the pooled MD ranged from -0.18 to 0.05, with all 95% CIs crossing the null line and all P values > 0.05, indicating that no single study had a decisive impact on the overall null result. The detailed results of the sensitivity analysis are presented in **Table 1**.

**Table 1:** Sensitivity analysis results.

Study excluded	Number of comparisons	Pooled MD	95% CI	I² (%)	*P*
None	5	-0.08	[–0.38, 0.22]	66%	0.61
Fernández et al., 2018 (1)	4	-0.18	[–0.47, 0.11]	60%	0.23
Fernández et al., 2018 (2)	4	-0.11	[–0.42, 0.21]	72%	0.51
Harris et al., 2024	4	-0.16	[–0.48, 0.16]	60%	0.32
Petrou et al., 2020 (3)	4	0.04	[–0.38, 0.46]	71%	0.86
Petrou et al., 2020 (4)	4	0.05	[–0.33, 0.43]	64%	0.81

MD, mean difference; CI, confidence interval; *I²*, inconsistency index. Sensitivity analysis was performed by omitting one study at a time. Pooled estimates were calculated using a random-effects model.

(1) Low-dose (1×10^6^ AdMSCs/kg) vs. placebo; (2) High-dose (4×10^6^ AdMSCs/kg) vs. placebo; (3) Intrathecal MSCs injection vs. sham; (4) Intravenous MSCs injection vs. sham.

### 3.6 Secondary outcomes

#### 3.6.1 MRI T2 lesion parameters

Only one RCT (Fernández et al., 2018) (39) reported changes in MRI T2 lesion volume and number from baseline to 12 months of follow-up. In this three-arm trial comparing low-dose (1×10^6^ cells/kg) AdMSCs, high-dose (4×10^6^ cells/kg) AdMSCs, and placebo, no significant differences were observed in T2 lesion volume or number changes between the MSC intervention groups and the placebo group. For T2 lesion volume, the mean change was +8.66 ± 16.32 cm³ in the low-dose group, +23.78 ± 58.47 cm³ in the high-dose group, and +16.42 ± 32.24 cm³ in the placebo group. For T2 lesion number, the mean change was +2.50 ± 8.44 in the low-dose group, +2.50 ± 5.46 in the high-dose group, and –1.30 ± 9.25 in the placebo group. Due to the extremely limited available data, no meta-analysis was performed for this outcome, and results are presented descriptively.

#### 3.6.2 Treatment-emergent adverse events)

**Figure 3:**
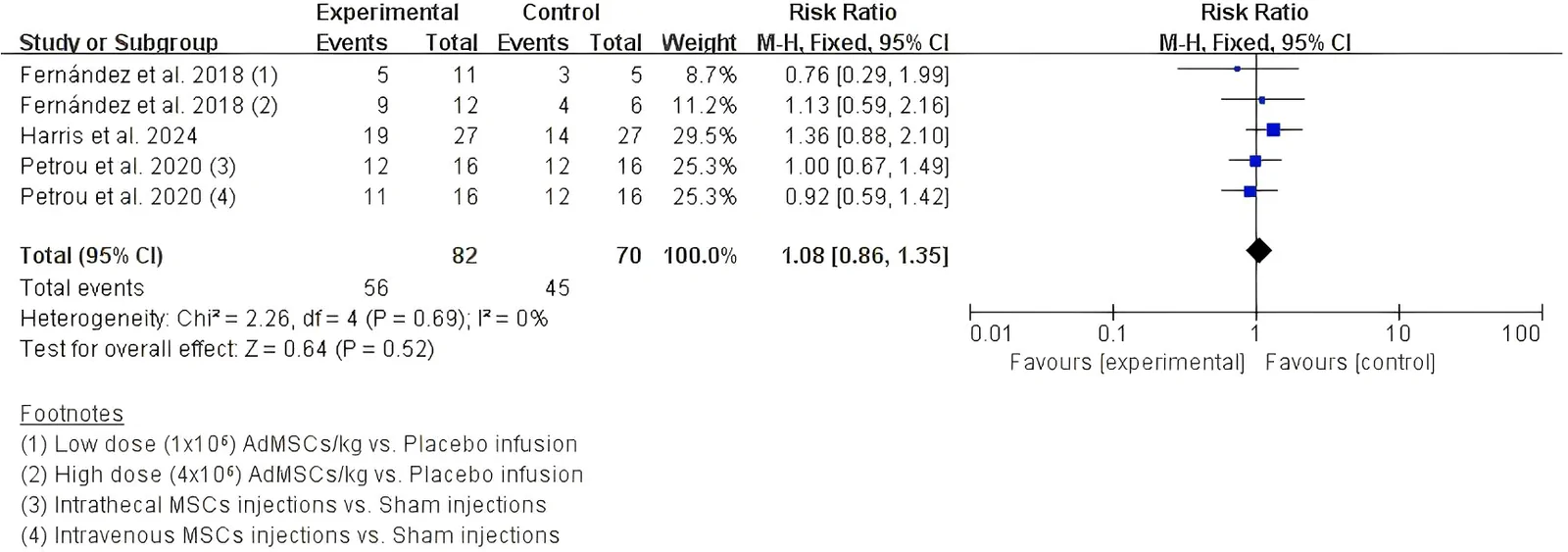
Forest plot of treatment-emergent adverse events (TEAEs) after MSC therapy compared with placebo.

All 3 included RCTs (contributing data from 5 treatment arms) reported the number of patients with TEAEs. Meta-analysis using a fixed-effects model showed that MSC transplantation did not significantly increase the risk of TEAEs in PMS patients, with a pooled RR of 1.08 (95% CI 0.86 to 1.35, Z = 0.64, *P* = 0.52). No between-study heterogeneity was observed (*I²* = 0%, *P* = 0.69). A total of 56 TEAEs were reported in 82 patients in the MSC group (68.3%), and 45 TEAEs in 70 patients in the control group (64.3%). The most commonly reported TEAEs were mild to moderate injection-related reactions, transient headache, low-grade fever, and fatigue, all of which were self-limiting without long-term sequelae. The forest plot for TEAEs is presented in **Figure 3**.

#### 3.6.3 Serious adverse events

**Figure 4:**
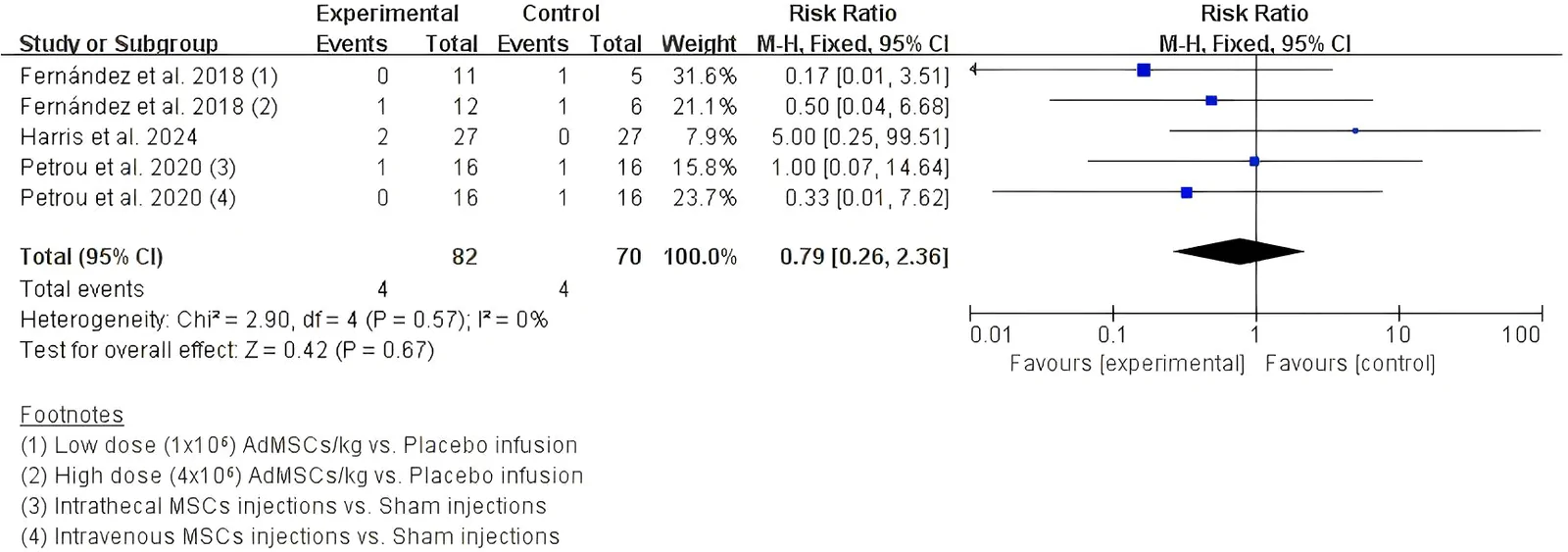
Forest plot of serious adverse events (SAEs) after MSC therapy compared with placebo.

All 3 included RCTs (contributing data from 5 treatment arms) reported the number of patients with SAEs. Meta-analysis using a fixed-effects model revealed no significant difference in the risk of SAEs between the MSC group and the control group, with a pooled RR of 0.79 (95% CI 0.26 to 2.36, Z = 0.42, *P* = 0.67). No between-study heterogeneity was observed (*I²* = 0%, *P* = 0.57). A total of 4 SAEs were reported in the MSC group (4.9% of 82 patients) and 4 SAEs in the control group (5.7% of 70 patients). None of the SAEs were deemed to be related to MSC transplantation by the original study investigators. The forest plot for SAEs is presented in **Figure 4**.

### 3.7 Certainty of evidence assessment

The GRADE summary of findings for all primary and secondary outcomes is presented in **Table 2**. The certainty of evidence was rated as very low for the primary outcome (change in EDSS score) due to high risk of bias, substantial inconsistency, serious imprecision, and suspected publication bias. For TEAEs, the certainty of evidence was rated as moderate, downgraded for high risk of bias. For SAEs, the certainty of evidence was rated as very low, due to very serious imprecision (extremely low event rate and wide 95% CI) and high risk of bias. Formal assessment of publication bias was not performed due to the limited number of included studies.

**Table 2:** Summary of findings.

Outcomes	No of patients		Effect		Certainty
	MSCs	Placebo	Relative (95% CI)	Absolute (95% CI)	
Change in EDSS score from baseline	59	53	–	MD 0.05 higher (0.33 lower to 0.43 higher)	Very low
Number of patients with TEAEs	56/82 (68.3%)	45/70 (64.3%)	RR 1.08 (0.86 to 1.35)	51 more per 1,000 (from 90 fewer to 225 more)	Moderate
Number of patients with SAEs	4/82 (4.9%)	4/70 (5.7%)	RR 0.79 (0.26 to 2.36)	12 fewer per 1,000 (from 42 fewer to 78 more)	Very low

EDSS, Expanded Disability Status Scale; TEAEs, treatment-emergent adverse events; SAEs, serious adverse events; MD, mean difference; RR, risk ratio; CI, confidence interval.

## 4 Discussion

In this systematic review and meta-analysis of exclusively RCTs, we found that MSC transplantation did not significantly improve EDSS scores in the overall unselected population of patients with PMS, despite substantial between-study heterogeneity. Individual study results varied: Petrou et al. (19) reported EDSS improvements in both IT and IV arms, Harris et al. (38) observed a numerical reduction with IT administration, whereas Fernández et al. (39) found no benefit with either low- or high-dose IV AdMSCs. The small number of studies precludes any formal exploration of heterogeneity sources. For safety outcomes, our results demonstrated that MSC transplantation has a favorable short-term safety and tolerability profile in PMS patients, with no increased risk of TEAEs or SAEs, consistent with prior studies in other autoimmune disease populations. The certainty of evidence was moderate for TEAEs, but very low for EDSS change and SAEs, indicating that future high-quality RCTs may substantially alter these effect estimates.

### 4.1 Comparison with prior systematic reviews and meta-analyses

Our findings are partially consistent with recently published meta-analyses, but provide critical novel insights by exclusively focusing on PMS patients and RCTs, eliminating the confounding effects of mixed MS subtypes and non-randomized study designs. A 2024 meta-analysis by Vaheb et al. (22), which pooled 30 studies (including non-randomized trials and mixed MS subtypes), reported that IT MSC administration led to a significant reduction in EDSS score, while IV delivery showed no benefit. Another 2026 meta-analysis by Maji et al. (23), which included 11 RCTs with mixed stem cell types (hematopoietic and mesenchymal) and mixed MS phenotypes, found a non-significant pooled EDSS reduction of 0.35 points favoring stem cell therapy. The smaller magnitude of the pooled effect size in our study (-0.08) is likely explained by our exclusive focus on PMS patients, who have more advanced neurodegeneration and less reversible disease than RRMS patients included in prior meta-analyses.

With regard to safety, our findings are in close agreement with the existing literature. Maji et al. (23) reported no difference in TEAE rates between stem cell and control groups (OR = 0.99, 95% CI 0.61–1.62, I² = 0%), and Vaheb et al. (22) concluded that MSC therapy is generally safe and well-tolerated, with adverse events mostly being mild and self-limiting. Importantly, our study is the first to provide a pooled safety estimate exclusively for PMS patients, confirming that MSC transplantation does not increase the risk of adverse events in this vulnerable population with advanced neurological disability.

### 4.2 Strengths of this study

This meta-analysis has several important strengths that distinguish it from prior published work:

1. Strict inclusion criteria: We exclusively enrolled RCTs, the highest level of clinical evidence, and focused specifically on patients with PMS, eliminating the confounding effects of non-randomized study designs and mixed MS subtypes that have limited prior meta-analyses.
2. Rigorous methodology: Our study was prospectively registered on PROSPERO, conducted in strict adherence to PRISMA 2020 guidelines and Cochrane standards, with independent dual screening, data extraction, and risk of bias assessment using the updated RoB 2 tool.
3. Rigorous exploration of heterogeneity: We systematically examined potential sources of between-study heterogeneity through sensitivity analysis and qualitative assessment, highlighting critical variables for future trial design.
4. Comprehensive evidence quality assessment: We performed a full GRADE assessment for all outcomes, providing a transparent evaluation of the certainty of evidence, which is critical for guiding clinical practice and future research.

### 4.3 Limitations

Several important limitations of this meta-analysis must be acknowledged:

1. Small number of included studies and limited sample size: Only 3 RCTs with a total of 143 patients were included in the quantitative synthesis, which resulted in insufficient statistical power, particularly for detecting rare adverse events such as SAEs, and limited our ability to perform meta-regression to quantitatively explore sources of heterogeneity.
2. Inability to perform subgroup analyses: According to Cochrane Handbook guidance, investigation of heterogeneity via subgroup analyses or meta-regression is unlikely to produce useful findings unless there is a substantial number of studies, with typical advice suggesting at least ten studies per characteristic modelled (24). With only three included RCTs, formal subgroup analyses were not feasible in this review, limiting our ability to quantitatively explore sources of heterogeneity such as administration route or disease activity.
3. Significant clinical heterogeneity across studies: The included studies varied widely in MSC source, dosage, administration route, patient baseline characteristics (disease activity, EDSS score, MS subtype), and follow-up duration, which may have affected the reliability of the pooled effect estimates, despite the use of random-effects models.
4. Limitations of the primary outcome measure: EDSS, the primary outcome used in all included studies, has well-documented limitations in PMS, particularly its heavy reliance on ambulatory function, which makes it insensitive to changes in upper limb function, cognition, fatigue, and other patient-relevant neurological domains. This may have led to an underestimation of the therapeutic effects of MSC transplantation on non-motor outcomes.
5. Lack of long-term follow-up data: All included studies had a maximum follow-up duration of 12 months, which is insufficient to assess the long-term durability of MSC therapeutic effects, as well as the risk of late-onset adverse events, including potential tumorigenicity.
6. Very low to moderate certainty of evidence: The GRADE assessment rated the evidence for the primary efficacy outcome and SAEs as very low, indicating that future high-quality RCTs are highly likely to have an important impact on the effect estimates and may alter our conclusions.
7. Inability to assess publication bias: With fewer than 10 included studies, we were unable to perform a funnel plot or statistical tests to assess publication bias, which is a potential limitation of all meta-analyses with a small number of included studies.

### 4.4 Implications for clinical practice and future research

Our findings have critical implications for both clinical practice and the design of future clinical trials of MSC transplantation for PMS:

1. The substantial heterogeneity observed across the included studies (I² = 66%) suggests that patient characteristics may influence treatment response. Future trials should systematically collect and report detailed baseline data—including disease activity status, EDSS score, and MS subtype—to enable investigation of potential effect modifiers.
2. The included studies employed both intrathecal and intravenous administration routes, contributing to clinical heterogeneity. Future trials should ideally compare different delivery routes head-to-head or standardize the route to reduce variability and clarify its impact on outcomes.
3. Standardization of MSC products is urgently needed: The heterogeneity of MSC products (source, manufacturing protocols, dosage, potency) is a major barrier to the clinical translation of MSC therapy. An international consensus on MSC manufacturing standards, potency assays, and release criteria for neurological indications is essential to reduce between-study heterogeneity and ensure consistent therapeutic effects.
4. Development of more sensitive and patient-relevant outcome measures: Future trials should move beyond EDSS as the sole primary outcome, and incorporate composite endpoints that include quantitative MRI metrics (brain atrophy, lesion load), CSF biomarkers of neuroaxonal damage (neurofilament light chain [NF-L]) and inflammation, neurophysiological tests, and patient-reported outcome measures (PROMs) to capture the full spectrum of treatment effects.
5. Larger, multicenter, high-quality RCTs are required: Given the very low certainty of current evidence, large-scale, multicenter, double-blind, placebo-controlled RCTs with rigorous design are urgently needed to validate the efficacy of MSC transplantation in PMS and to identify factors associated with treatment response. These trials should include predefined subgroup analyses, long-term follow-up (≥ 24 months), and serial biomarker monitoring to elucidate the mechanisms of action of MSC therapy in PMS.
6. Exploration of combination therapeutic strategies: Future preclinical and clinical studies should explore the efficacy of MSC transplantation in combination with other DMTs (e.g., anti-CD20 monoclonal antibodies) or remyelination-promoting agents, to synergistically target both inflammation and neurodegeneration, the two core pathophysiological hallmarks of PMS.

## 5 Conclusion

This systematic review and meta-analysis of RCTs demonstrates that MSC transplantation has a favorable short-term safety and tolerability profile in patients with PMS, with no increased risk of TEAEs or SAEs. However, current aggregate evidence does not support a significant overall beneficial effect of MSC transplantation on improving EDSS scores in unselected PMS patients. The included studies showed heterogeneous results, with some trials suggesting potential benefit while others did not.

Notably, this meta-analysis has several important limitations that should be considered when interpreting the results, including the small number of included studies and limited sample size, inability to perform subgroup analyses to identify potential effect modifiers, short follow-up duration, and very low to moderate certainty of evidence for key outcomes.

Given the very low to moderate certainty of current evidence, these findings should be interpreted with caution. Further large-scale, multicenter, high-quality RCTs with standardized MSC manufacturing protocols are urgently warranted to validate the clinical efficacy of MSC transplantation for PMS and to identify the optimal delivery route and responsive patient population.

## Data Availability

The original contributions presented in the study are included in the article/**Supplementary Material**. Further inquiries can be directed to the corresponding author.
