# Correction: Efficacy and safety of mesenchymal stem cell transplantation for progressive multiple sclerosis: a systematic review and meta-analysis of randomized controlled trials with clinical implications for patient stratification and treatment optimization

**DOI:** 10.3389/fimmu.2026.1983818

**Published:** 2026-09-11

**Authors:** Honghao Guo, Liuting Zeng, Lingyun Sun

**Affiliations:** 1 Department of Rheumatology and Immunology, Nanjing Drum Tower Hospital, Clinical College of Nanjing Medical University, Nanjing, Jiangsu, China; 2 Department of Rheumatology and Immunology, Nanjing Drum Tower Hospital, Chinese Academy of Medical Sciences & Peking Union Medical College, Nanjing, China

**Keywords:** mesenchymal stem cells, meta-analysis, progressive multiple sclerosis, randomized controlled trials, safety

The order of authors in the author list of the published paper was erroneously given as:

“Honghao Guo¹, Lingyun Sun¹,²*, Liuting Zeng²”

The correct author list reads:

“Honghao Guo¹, Liuting Zeng² and Lingyun Sun¹,²*”

The original version of this article has been updated.

