# Supplementary material for: Efficacy and safety of mesenchymal stem cell transplantation for progressive multiple sclerosis: a systematic review and meta-analysis of randomized controlled trials with clinical implications for patient stratification and treatment optimization
Source: Front Immunol. 2026 Jul 22;17:1861624. doi: 10.3389/fimmu.2026.1861624 (PMC13437468; doi:10.3389/fimmu.2026.1861624)
Supplement: Supplementary file 1 [file Image1.pdf]

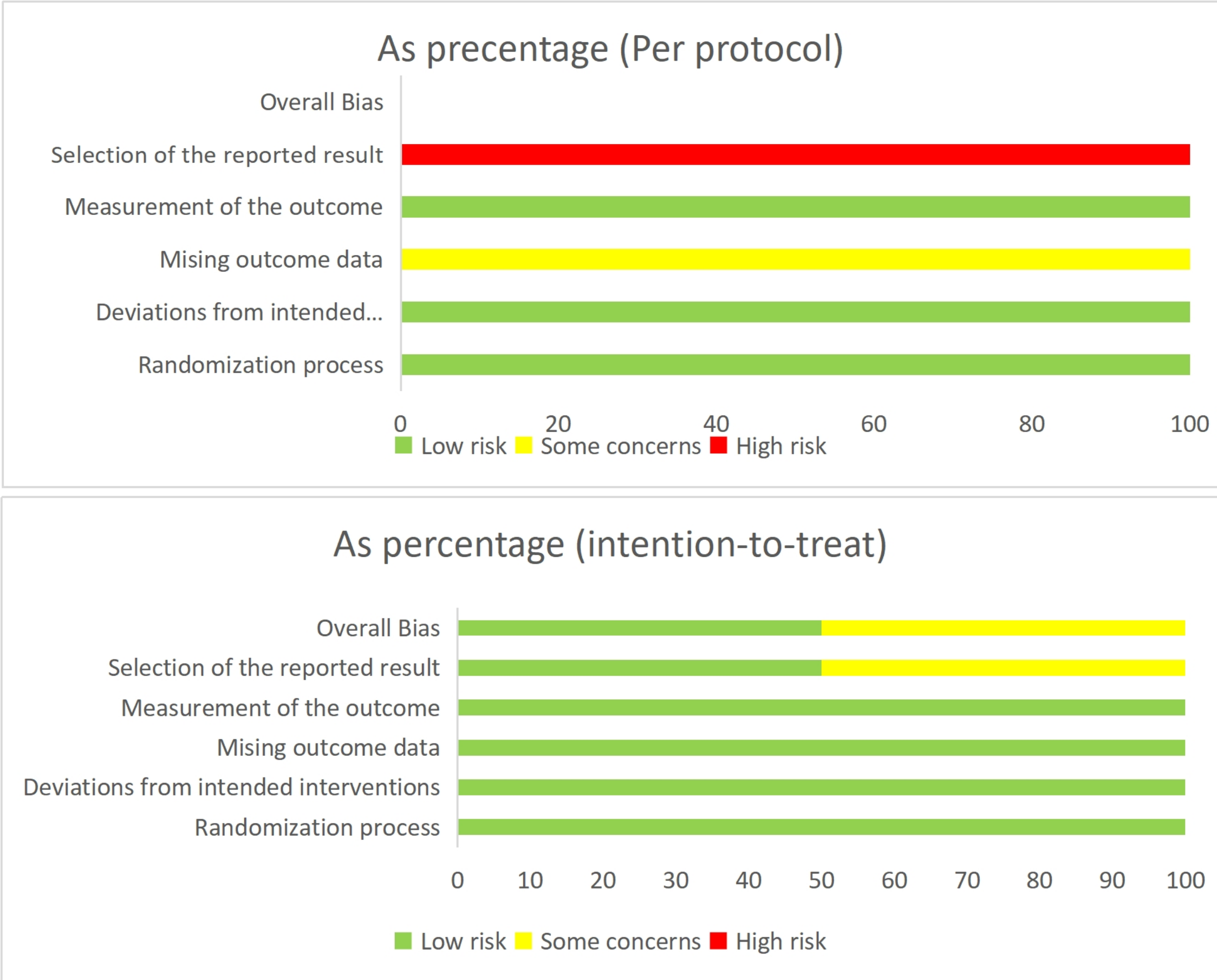

Figure S1 Risk of bias graph (RoB 2)

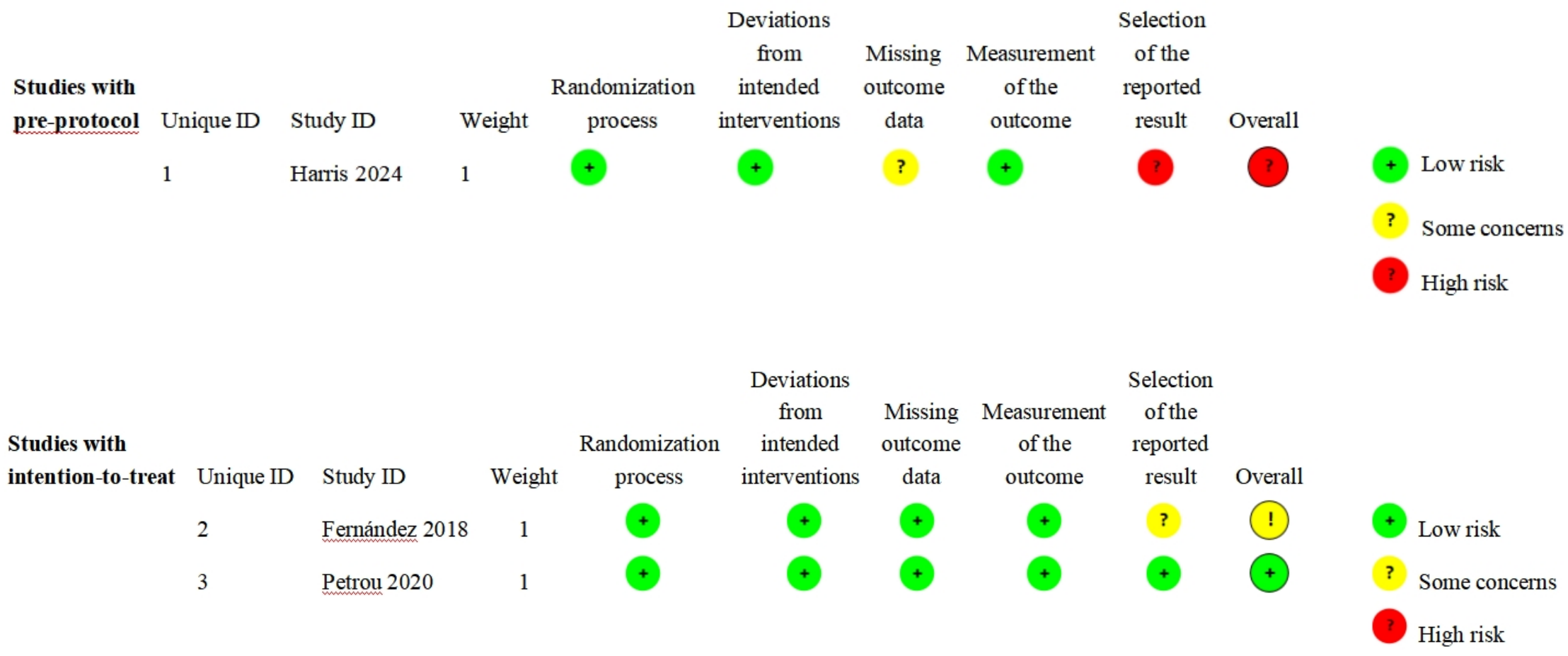

Figure S2 Risk of bias summary (RoB 2)
