# Supplementary material for: Efficacy and safety of mesenchymal stem cell transplantation for progressive multiple sclerosis: a systematic review and meta-analysis of randomized controlled trials with clinical implications for patient stratification and treatment optimization
Source: Front Immunol. 2026 Jul 22;17:1861624. doi: 10.3389/fimmu.2026.1861624 (PMC13437468; doi:10.3389/fimmu.2026.1861624)
Supplement: Supplementary file 2 [file Table1.docx]

**Table S1.** Search Strategies for Pubmed and Embase

| **PubMed** | (((((((((("Multiple Sclerosis"[Mesh]) OR (Sclerosis, Multiple[Title/Abstract])) OR (MS (Multiple Sclerosis[Title/Abstract]))) OR (Sclerosis, Disseminated[Title/Abstract])) OR (Disseminated Sclerosis[Title/Abstract])) OR (Multiple Sclerosis, Acute Fulminating[Title/Abstract])) OR (primary progressive multiple sclerosis[Title/Abstract])) OR (PPMS[Title/Abstract])) OR (secondary progressive multiple sclerosis[Title/Abstract])) OR (SPMS[Title/Abstract]))  AND  (((((((((((((((((((((((((((((((((((((((((((((((((((((((((((("Stem Cells"[Mesh]) OR (Cells, Stem[Title/Abstract])) OR (Cell, Stem[Title/Abstract])) OR (Stem Cell[Title/Abstract])) OR (Mother Cells[Title/Abstract])) OR (Cell, Mother[Title/Abstract])) OR (Cells, Mother[Title/Abstract])) OR (Mother Cell[Title/Abstract])) OR (Progenitor Cells[Title/Abstract])) OR (Cell, Progenitor[Title/Abstract])) OR (Cells, Progenitor[Title/Abstract])) OR (Progenitor Cell[Title/Abstract])) OR (Colony-Forming Unit[Title/Abstract])) OR (Colony Forming Unit[Title/Abstract])) OR (Colony-Forming Units[Title/Abstract])) OR (Colony Forming Units[Title/Abstract])) OR (mesenchymal stem cells[Title/Abstract])) OR (MSC[Title/Abstract])) OR (Stem Cell, Mesenchymal[Title/Abstract])) OR (Mesenchymal Stem Cell[Title/Abstract])) OR (Stem Cells, Mesenchymal[Title/Abstract])) OR (Mesenchymal Stromal Cells[Title/Abstract])) OR (Mesenchymal Stromal Cell[Title/Abstract])) OR (Stromal Cell, Mesenchymal[Title/Abstract])) OR (Stromal Cells, Mesenchymal[Title/Abstract])) OR (Wharton Jelly Cells[Title/Abstract])) OR (Wharton's Jelly Cells[Title/Abstract])) OR (Wharton's Jelly Cell[Title/Abstract])) OR (Whartons Jelly Cells[Title/Abstract])) OR (Bone Marrow Stromal Cells[Title/Abstract])) OR (Bone Marrow Stromal Cell[Title/Abstract])) OR (Bone Marrow Stromal Cells, Multipotent[Title/Abstract])) OR (Multipotent Bone Marrow Stromal Cell[Title/Abstract])) OR (Multipotent Bone Marrow Stromal Cells[Title/Abstract])) OR (Bone Marrow Stromal Stem Cells[Title/Abstract])) OR (Mesenchymal Progenitor Cell[Title/Abstract])) OR (Mesenchymal Progenitor Cells[Title/Abstract])) OR (Progenitor Cell, Mesenchymal[Title/Abstract])) OR (Progenitor Cells, Mesenchymal[Title/Abstract])) OR (Multipotent Mesenchymal Stromal Cells[Title/Abstract])) OR (Mesenchymal Stromal Cells, Multipotent[Title/Abstract])) OR (Multipotent Mesenchymal Stromal Cell[Title/Abstract])) OR (Bone Marrow Mesenchymal Stem Cells[Title/Abstract])) OR (Bone Marrow Mesenchymal Stem Cell[Title/Abstract])) OR (Adipose-Derived Mesenchymal Stem Cells[Title/Abstract])) OR (Adipose Derived Mesenchymal Stem Cells[Title/Abstract])) OR (Adipose-Derived Mesenchymal Stromal Cells[Title/Abstract])) OR (Adipose Derived Mesenchymal Stromal Cells[Title/Abstract])) OR (Mesenchymal Stem Cells, Adipose-Derived[Title/Abstract])) OR (Mesenchymal Stem Cells, Adipose Derived[Title/Abstract])) OR (Adipose Tissue-Derived Mesenchymal Stromal Cell[Title/Abstract])) OR (Adipose Tissue Derived Mesenchymal Stromal Cell[Title/Abstract])) OR (Adipose Tissue-Derived Mesenchymal Stromal Cells[Title/Abstract])) OR (Adipose Tissue Derived Mesenchymal Stromal Cells[Title/Abstract])) OR (Adipose Tissue-Derived Mesenchymal Stem Cell[Title/Abstract])) OR (Adipose Tissue Derived Mesenchymal Stem Cell[Title/Abstract])) OR (Adipose Tissue-Derived Mesenchymal Stem Cells[Title/Abstract])) OR (Adipose Tissue Derived Mesenchymal Stem Cells[Title/Abstract])) OR (Adipose-Derived Mesenchymal Stem Cell[Title/Abstract])) OR (Adipose Derived Mesenchymal Stem Cell[Title/Abstract]))  AND  ("Placebos"[Mesh] OR placebo* OR saline OR "disease-modifying therapy" OR DMT OR "interferon beta" OR "glatiramer acetate" OR "natalizumab" OR "fingolimod")  AND  ("Treatment Outcome"[Mesh] OR efficacy OR "Expanded Disability Status Scale" OR EDSS OR "MRI T2 lesions" OR safety OR "adverse effects" OR "adverse events" OR "treatment-emergent adverse events") |
| --- | --- |
| **EMBASE** | ('multiple sclerosis':ti,ab,kw OR 'chariot disease':ti,ab,kw OR 'disseminated sclerosis':ti,ab,kw OR 'insular sclerosis':ti,ab,kw OR (ms:ti,ab,kw AND 'multiple sclerosis':ti,ab,kw) OR 'sclerosis multiplex':ti,ab,kw OR 'sclerosis, disseminated':ti,ab,kw OR 'sclerosis, insular':ti,ab,kw OR 'sclerosis, multiple':ti,ab,kw)  AND  ('stem cell':ti,ab,kw OR 'cell, stem':ti,ab,kw OR 'precursor cell':ti,ab,kw OR 'progenitor cell':ti,ab,kw OR 'stem cells':ti,ab,kw OR 'mesenchymal stem cell':ti,ab,kw OR 'mesenchymal progenitor cell':ti,ab,kw OR 'mesenchymal stem cells':ti,ab,kw OR 'stem cell, mesenchymal':ti,ab,kw)  AND  ('placebo'/exp OR placebo* OR saline OR 'disease modifying therapy' OR dmt OR 'interferon beta' OR 'glatiramer acetate' OR 'natalizumab' OR 'fingolimod')  AND  ('treatment outcome'/exp OR efficacy OR 'expanded disability status scale' OR edss OR 'mri t2 lesion*' OR safety OR 'adverse drug reaction'/exp OR 'adverse event*' OR 'treatment emergent adverse event*') |
